# Vision does not always help stroke survivors compensate for impaired limb position sense

**DOI:** 10.1186/s12984-019-0596-7

**Published:** 2019-10-30

**Authors:** Troy M. Herter, Stephen H. Scott, Sean P. Dukelow

**Affiliations:** 1 Department of Exercise Science, University of South Carolina, Columbia, SC USA; 2 Centre for Neuroscience Studies, Queen’s University, Kingston, Ontario Canada; 3 Department of Anatomy and Cell Biology, Queen’s University, Kingston, Ontario Canada; 4 School of Medicine, Queen’s University, Kingston, Ontario Canada; 5 Hotchkiss Brain Institute, University of Calgary, Calgary, Alberta Canada; 6 Department of Clinical Neurosciences, University of Calgary, 1403 29th St NW, Foothills Medical Centre, South Tower–Room 905, Calgary, AB T2N2T9 Canada

**Keywords:** Stroke, Position sense, Proprioception, Vision, Robotics, Assessment

## Abstract

**Background:**

Position sense is commonly impaired after stroke. Traditional rehabilitation methods instruct patients to visualize their limbs to compensate for impaired position sense.

**Objective:**

Our goal was to evaluate how the use of vision influences impaired position sense.

**Methods:**

We examined 177 stroke survivors, an average of 12.7 days (+/− 10 days (SD)) post-stroke, and 133 neurologically-intact controls with a robotic assessment of position sense. The robot positioned one limb (affected) and subjects attempted to mirror-match the position using the opposite limb (unaffected). Subjects completed the test without, then with vision of their limbs. We examined three measures of position sense: variability (*Var*), contraction/expansion (*C/E*) and systematic shift (*Shift*). We classified stroke survivors as having full compensation if they performed the robotic task abnormally without vision but corrected performance within the range of normal with vision. Stroke survivors were deemed to have partial compensation if they performed the task outside the range of normal without and with vision, but improved significantly with vision. Those with absent compensation performed the task abnormally in both conditions and did not improve with vision.

**Results:**

Many stroke survivors demonstrated impaired position sense with vision occluded [*Var*: 116 (66%), *C/E*: 91 (51%), *Shift*: 52 (29%)]. Of those stroke survivors with impaired position sense, some exhibited full compensation with vision [*Var*: 23 (20%), *C/E*: 42 (46%), *Shift*: 32 (62%)], others showed partial compensation [*Var*: 37 (32%), *C/E*: 8 (9%), *Shift*: 3 (6%)] and many displayed absent compensation (*Var*: 56 (48%), *C/E*: 41 (45%), *Shift*: 17 (33%)]. Stroke survivors with an affected left arm, visuospatial neglect and/or visual field defects were less likely to compensate for impaired position sense using vision.

**Conclusions:**

Our results indicate that vision does not help many stroke survivors compensate for impaired position sense, at least within the current paradigm. This contrasts with historical reports that vision helps compensate for proprioceptive loss following neurologic injuries.

## Introduction

Most studies of upper-limb stroke rehabilitation focus on treating motor impairments to improve functional outcomes. As a result, sensory impairments are often ignored despite being both common and correlated with poor functional recovery [1, 2]. Proprioception, our awareness of limb position and movement without the use of vision [3], is a key sensory modality used to plan, control, and learn motor skills [4–7]. Proprioception includes two major subcomponents: position sense (knowledge of limb position) and kinesthesia (knowledge of limb movement) [8]. Approximately 50–60% of stroke survivors have impaired position sense and/or kinesthesia [9–12], and these impairments are associated with poor functional outcomes independent of impaired motor function [13, 14].

A commonly used method for rehabilitation of impaired proprioception is to instruct patients to observe their affected limb during task-oriented (functional) practice [10, 15–17]. The premise is that vision allows patients to compensate for proprioceptive impairments and to potentially encourage proprioceptive recovery. Although this method is widely applied in clinical practice, there is little evidence that it produces improvements in proprioception and functional outcomes after stroke. Clinical implementation appears to stem from studies of patients with peripheral nerve injuries [18] and, over the years, this has been anecdotally accepted as a viable treatment for impaired proprioception following stroke. Notably, we found only a single case study in which a single subject with chronic stroke compensated for impaired position sense using vision [19]. There is, however, indirect evidence that observing the affected limb during task-oriented practice might produce improvements in proprioception that mediate better functional outcomes. First, integration of vision and proprioception contribute to perception of hand position [20] and to sensorimotor planning [21], control [22], adaptation [23], and learning [24]. Second, practicing reaching movements with vision of the limbs produces improvements in position sense in normal human subjects [25]. Third, healthy adults exhibit improvements in position sense during motor learning using alternating blocks with and without vision [26].

Despite the widespread assumption that vision can help to improve or compensate for impaired position sense, it appears that this has never been quantified in a group of stroke survivors. The first objective of this study was to determine the extent to which stroke survivors can use vision to compensate for impaired position sense. Our second objective was to examine the extent to which stroke side, vascular territory, and perceptual impairments are associated with differences using vision to compensate for impaired position sense. Our third objective was to examine the extent to which greater compensation for impaired position sense is associated with increased functional ability. We used recent innovations in upper-limb robotics [11, 12, 27, 28] to accurately and reliably examine the ability of stroke survivors to compensate for impaired position sense with vision. We hypothesized that most stroke survivors would exhibit normal position sense with vision, and larger improvements in position sense with vision would be associated with increased functional independence.

## Methods

### Subjects

Stroke survivors were recruited from inpatient stroke units at the Foothills Medical Centre and Dr. Vernon Fanning Care Centre in Calgary, Alberta, Canada. Nondisabled controls were recruited from the community.

Subjects were included if they were 18 years of age or older and could understand the instructions required to complete the assessments. Subjects were excluded if they had: visual acuity worse than 20/50 (corrected), upper-limb orthopedic problems or other neurologic disorders. No patient reported a history of significant cognitive deficits (e.g., Alzheimer’s disease, vascular dementia).

Characteristics of stroke, including type, side, vascular territory and days since stroke, were obtained from clinical case histories and neuroimaging (magnetic resonance imaging or computed tomography). Rather than classifying subjects based on the side of their stroke, they were classified based on the more affected side of their body (left-affected or right-affected). This provided a more robust classification for interpreting data because some brainstem and cerebellar strokes affect the ipsilateral side of the body. Vascular territories were grouped into five broad territories, including middle cerebral artery (MCA), posterior cerebral artery (PCA), anterior cerebral artery (ACA), brainstem vasculature, and cerebellar vasculature.

### Robotic assessment of position sense

#### Arm-position matching task

**Fig. 1:**
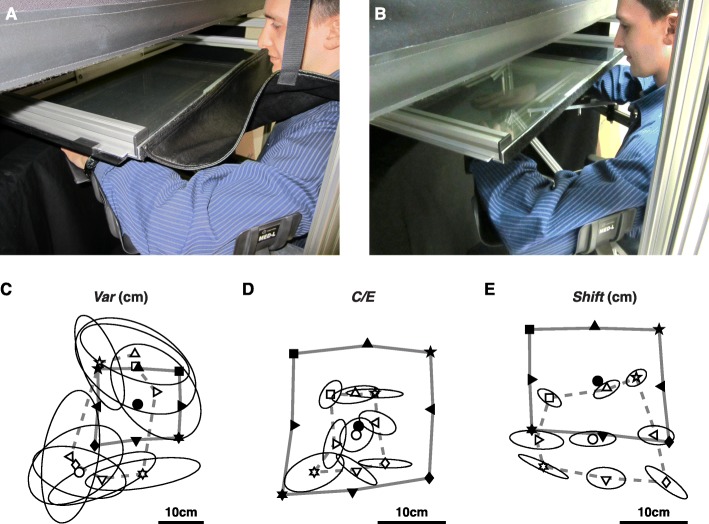
Robotic apparatus, measures of position sense, and exemplar task performance. **a**, Occluded vision condition, showing a subject in the robotic apparatus with the shields closed and fabric cover in place to prevent vision of his arms and hands. **b**, Normal vision condition, showing a subject in the robotic apparatus with the shields pulled back and the fabric cover removed to allow full vision of his arms and hands. **c-e**, Representative impairments on our measures of position sense: **c**, Variability (*Var*, right), **d**, Contraction/Expansion (*C/E*, middle), and **e**, *Shift* (right). The robot passively moved the affected arm to each of nine spatial locations (filled symbols) and the subjects actively moved their less affected arm to mirror-match each spatial location (open symbols). Each plot shows the overlap between the passive and active hands after mirror transforming data from the left side to the right side of the workspace. Ellipses around open symbols represent one standard deviation. Areas enclosed by the thick grey lines show the matching areas of passive (solid) and active (dashed) arms

We used a KINARM exoskeleton robot (BKIN Technologies Ltd., Kingston, ON) [29] to assess upper-limb position sense with a previously described arm-position matching task (Fig. 1) [11, 30–32]. In brief, the robot moved one arm (passive arm) to one of nine spatial locations separated by 10 cm (Fig. 1). After the robot finished moving the passive arm, subjects actively mirror-matched the position with the opposite arm (active arm). For stroke survivors, the robot moved the affected arm and they matched with the opposite, less-affected arm. Subjects were permitted as much time as needed to mirror-match the target position before informing the experimenter to initiate the next trial. This ensured that measures of position sense were minimally affected by potential motor impairments of the less-affected arm. Subjects completed six trials to each of the nine targets in randomized blocks for a total of 54 trials. Control subjects were tested on both arms.

#### Occluded vision

Subjects performed the arm-position matching task with their arms, hands, and body completely occluded from vision by an opaque shield mounted above the hands and a fabric cover placed between the shield and each subject’s neck (Fig. 1a).

#### Normal vision

Subjects repeated the arm-position matching task after removing the shield and fabric cover (Fig. 1b), which allowed full vision of the arms, hands, and body. The task was always performed with occluded vision before normal vision.

#### Measures of position sense

As in previous studies [11, 32], we quantified three measures of position sense: 1) Variability (*Var*) is the mean trial-to-trial variability of the active hand as measured in centimetres [11]. *Var* attempts to capture a subject’s accuracy in performing the task. *Var* was calculated by finding the standard deviation of the active hand’s position for each target location, then calculating the mean of standard deviations for all target locations in the x coordinate (*Varx*) and y coordinate (*Vary*), and the linear variability for both coordinates combined (*Var*):

$$ Var=\sqrt{Var{x}^2+ Var{y}^2} $$

2) Contraction/Expansion (*C/E*) describes the area of the workspace matched by the active hand relative to that of the passive hand. This is a parameter that examines issues with spatial awareness across the entire target set and we have previously reported that some individuals with stroke have difficulty perceiving their workspace so will present with significantly contracted or expanded representations of their workspace [11]. *C/E* was determined by finding the area spanned by the active hand for the 8 peripheral targets, then normalizing it to the total spatial area spanned by the passive hand. As a ratio, *C/E* is unitless and values typically range between 0 and approximately 2.

3) Systematic Shifts (*Shift*) describes the mean error between the passive and active hands and is measured in centimetres. *Shift* examines for potential systematic bias of the subject’s ability to match. *Shift* was calculated by first obtaining systematic shifts in the *x* (*Shiftx*), then in the *y* (*Shifty*) and then the combination (*Shift*).

$$ Shift=\sqrt{Shift{x}^2+ Shift{y}^2} $$

Figure 1c, d and e illustrate exemplar impairments of *Var*, *C/E* and *Shift*, respectively. For this study, *C/E* was normalized by control performance to create positive scores for both contraction and expansion. Values between 0 and 1 indicate performance within 95% of controls, whereas values greater than 1 indicate performance outside this range.

### Clinical assessment

A trained stroke physiatrist, neurologist, or therapist completed a standardized, clinical assessment, which included a broad range of evaluations that examined functional abilities and perceptual, motor, and cognitive impairments. Functional abilities were assessed using the Functional Independence Measure (FIM) [33]. We assessed impairments of position sense, without vision, using the Thumb Localizer Test (TLT) [34], visual acuity using a Snellen Eye Chart, visual field defects using the confrontation technique [35], and visuospatial neglect using the conventional subsets of the Behavioral Inattention Test (BIT) [36]. We assessed impairments affecting several features of motor behavior, including hand dexterity using the Purdue Pegboard (PPB) (Lafayette Instrument Co., Lafayette, IN, USA), arm and hand impairment using the Chedoke-McMaster Stroke Assessment (CMSA) impairment inventories [37], and spasticity at the elbow using the Modified Ashworth Scale (MAS) [38]. Cognitive function was assessed with the Montreal Cognitive Assessment (MoCA) [39]. We assessed handedness before stroke with the Modified Edinburgh Handedness Inventory [40]. Control subjects did not complete the FIM, TLT, BIT, CMSA, MAS, or MoCA.

### Analysis

We examined differences in demographic and clinical data between subgroups of stroke survivors using Kolmogorov-Smirnov (KS) tests for continuous data (age, days since stroke, FIM, FIMsc, BIT, PPB, MoCA) and chi-squared tests for categorical and discrete data (sex, handedness, stroke type, TLT, visuospatial neglect, visual field defects, CMSA, MAS).

To examine the extent to which stroke survivors used vision to compensate for impaired position sense, we initially identified stroke survivors with impaired position sense. To do this we established normative reference ranges from our controls. First, we ensured there were no differences in control subject in performance between the first and second hand performing the task in the occluded vision condition (which could potentially arise from learning effects) on any of the parameters using a paired t-test (*Varp* = 0.29, *C/Ep* = 0.75, *Shiftp* = 0.64). Next we used regression models from the control data to establish normative reference ranges specific to age, sex and test-hand of each stroke survivor [32]. We were able to use the average measures from both hands of the controls for our normal range, except for *C/E* in the Normal Vision condition, where we noted a significant difference in the performance of the dominant and non-dominant arm of controls and made our comparisons accordingly. The normative reference ranges were used to identify stroke survivors who were *Normal* (inside 95% normative reference range) or *Impaired* (outside 95% normative reference range) on each measure. We then examined relationships between visual conditions using Pearson correlations (Occluded versus Normal) and Fisher’s tests of independence categorized by visual condition (Occluded or Normal) and performance (Normal or Impaired). For those who were Impaired in both conditions, we also used paired *t*-tests to identify individuals who significantly improved with normal vision (*p* < 0.05). Finally, we categorized individuals based on their compensation with normal vision: 1) *Normal* (normal in both conditions), 2) *Full* compensation (Impaired with occluded vision but Normal with normal vision), 3) *Partial* compensation (Impaired in both conditions, but significantly better with normal vision), 4) *Inverse* compensation (Normal with occluded vision but Impaired with normal vision), and 5) *Absent* compensation (Impaired in both conditions, and did not significantly improve with normal vision).

To examine the relationships between compensation with normal vision and clinical measures of functional ability and impairment, we performed Spearman correlations between robotic measures and continuous clinical measures (FIM, BIT, PPB, MoCA). We considered Spearman correlations as “weak” from 0.10 to 0.30, “moderate” from 0.30 to 0.50, and “strong” from 0.5 to 1.0 [41]. We also performed Fisher’s tests between performance categories (Normal or Impaired) based on robotic measures and discrete clinical measures (TLT, CMSA). Individuals with TLT scores of 0 were classified as Normal and those with TLT scores greater than 0 were classified as Impaired. Individuals with CMSA scores of 7 were classified as Normal and those with CMSA scores less than 7 were classified as Impaired. Finally, we used analysis of variance (ANOVA) to examine differences between compensation groups (Normal, Full, Partial, Absent) on continuous clinical measures (FIM, BIT, PPB, MoCA).

## Results

### Subject characteristics

We recruited 177 unilateral stroke survivors and 130 controls for the study (Table 1). Our sample of stroke survivors included a typical distribution of vascular territories observed in rehabilitation (Table 2). We did not find significant differences between left- and right-affected stroke survivors in their age, sex, handedness, stroke type, days since stroke, FIM, TLT, field defects, PPB, CMSA, MAS, and MoCA (all *p* ≥ 0.05). However, we found significant differences in BIT scores (*p* < 0.05). Controls were recruited from a relatively uniform distribution of ages from 20 to 90 (Table 3).

**Table 1:** Demographic and clinical information

Measure	Stroke Survivors		Control Subjects
	Left-Affected {*n* = 108}	Right-Affected {*n* = 69}	{*n* = 130}
Age	64 (20–89)	62 (21–90)	56 (20–88)
Sex	78 M, 30 F	41 M, 28 F	67 M, 63 F
Handedness	99 R, 4 L, 5 A	60 R, 5 L, 4 A	112 R, 12 L, 6 A
Stroke type	87 Isc, 21 Hem	59 Isc, 10 Hem	–
Days since stroke	10 (1–55)	11 (2–52)	–
FIM	96 (35–126)	103 (40–126)	–
TLT affected arm [0–3]	[9, 22, 31, 46]	[4, 10, 17, 38]	–
BIT	138 (51–146)	143 (58–146)	–
Visual field defects	24 Y, 84 N	8 Y, 61 N	–
PPB affected	3 (0–13)	4 (0–15)	–
PPB less affected	10 (4–16)	10 (5–16)	14 (8–18)
CMSA affected arm [1–7]	[4, 9, 11, 15, 22, 31]^a^	[4, 6, 10, 13, 20]	–
CMSA affected hand [1–7]	[6, 8, 14, 18, 24, 28]^b^	[5, 6, 9, 11, 16, 17]	–
CMSA less affected arm [1–7]	[0, 0, 0, 0, 4, 21, 82]^a^	[0, 0, 0, 0, 0, 6, 63]	–
CMSA less affected hand [1–7]	[0, 0, 0, 0, 0, 34, 72]^b^	[0, 0, 0, 0, 3, 18, 48]	–
MAS affected arm [0–4]	[77, 15, 7, 8, 0, 0]^a^	[52, 9, 4, 1, 1, 0]^d^	–
MAS less affected arm [0–4]	[104, 3, 0, 0, 0, 0]^a^	[67, 0, 0, 0, 0, 0]^d^	–
MoCA	24 (13–30)^c^	24 (8–29)^e^	–

Age, days since stroke, Functional Independence Measure (FIM), Behavioral Inattention Test (BIT), Purdue Peg Board (PPB), and Montreal Cognitive Assessment (MoCA) scores are provided as the median and range (). Thumb Localizing Test (TLT), Chedoke-McMaster Stroke Assessment (CMSA) and Modified Ashworth Scores (MAS) provide number of subjects in each category []. For example, 6 values for MAS correspond to number of subjects that scored [0, 1, 1+, 2, 3, 4]. TLT values provide scores in which stroke survivors localized their affected thumb using their less affected hand. Individuals with hemispatial neglect were categorized from BIT scores (BIT< 129) [24]. Stoke types included ischemic (Isc) and hemorrhagic (Hem). Some clinical assessments were not obtained from all stroke survivors: ^a^ = 107, ^b^ = 106, ^c^ = 104, ^d^ = 67, ^e^ = 68

**Table 2:** Stroke Territories

	Subgroup	# Subjects {*n* = 177}
Single Territory	MCA	117
	PCA	22
	ACA	6
	Basilar	6
	Vertebral	4
	Pontine	4
	PICA	3
	Internal Carotid	2
	Anterior Choroidal	1
Multiple Territories	MCA, ACA	5
	MCA, PCA	3
	MCA, ACA, PCA	1
Other	Unknown	2
	Other	1

**Table 3:** Control subject demographics

Sex	Age Range							
	20–29	30–39	40–49	50–59	60–69	70–79	80–89	All Ages
Male	7	10	7	9	14	14	6	67
Female	10	10	10	10	11	10	2	63
Both	17	20	17	19	25	24	8	130

### Examining the ability to compensate for impaired position sense with vision

#### Exemplar subjects

**Fig. 2:**
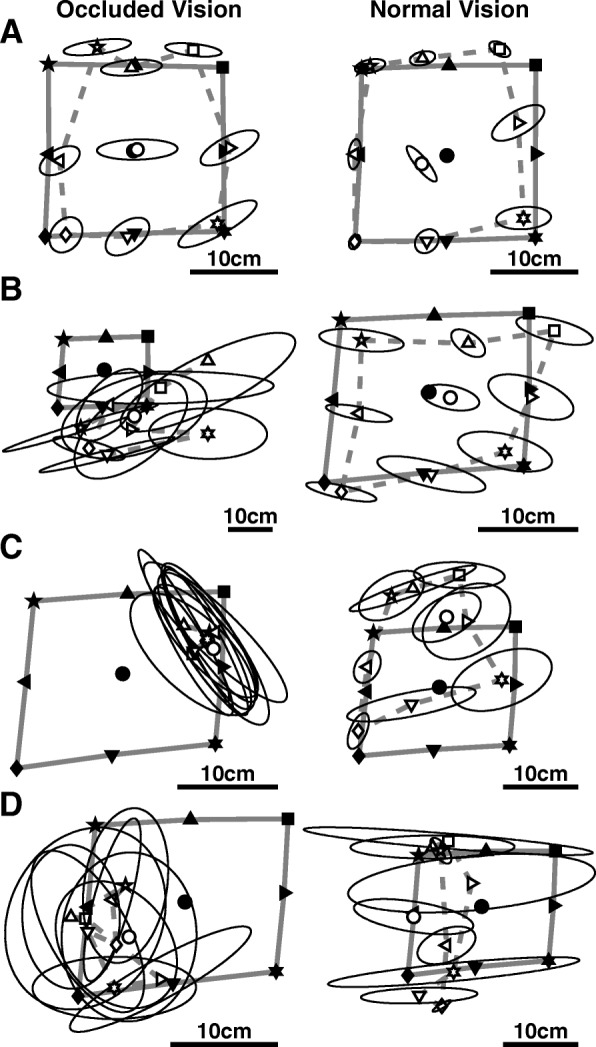
**a**-**d**, representative task performance with occluded (left) and normal (right) vision. **a**, Control subject showing Normal performance with occluded vision (*Var* = 3.8 cm, *C/E* = 0.87, *Shift* = 0.7 cm) and normal vision (*Var* = 2.7 cm, *C/E* = 0.92, *Shift* = 1.9 cm). **b**, Stroke survivors showing Full compensation (Impaired with occluded vision: *Var* = 18.6 cm, *C/E* = 0.08, *Shift* = 12.7 cm; Normal with normal vision: *Var* = 3.7 cm, *C/E* = 0.74, *Shift* = 3.7 cm). **c**, Stroke survivor showing Partial compensation (Impaired with occluded vision: *Var* = 11.5 cm, *C/E* = 0.09, *Shift* = 8.8 cm; impaired but significantly improved with normal vision: *Var* = 7.1 cm, *C/E* = 0.73, *Shift* = 6.6 cm). **d**, Stroke survivor showing Absent compensation (Impaired with occluded vision: *Var* = 11.5 cm, *C/E* = 0.43, *Shift* = 7.4 cm; Impaired with normal vision: *Var* = 11.2 cm, *C/E* = 0.42, *Shift* = 5.6 cm)

To determine the extent to which stroke survivors can use vision to compensate for impaired position sense, we compared position sense with occluded and normal vision. Figure 2 shows representative position matching of a control and three stroke survivors in both conditions. The control exhibited modest improvement between conditions (Fig. 2a), which was typical of controls. In contrast, the stroke survivors exhibited three distinct patterns of compensation. All three were Impaired with occluded vision, but the first shows Full compensation (Fig. 2b), the second Partial compensation (Fig. 2c), and the third Absent compensation (Fig. 2d).

#### Group data - Variability

**Fig. 3:**
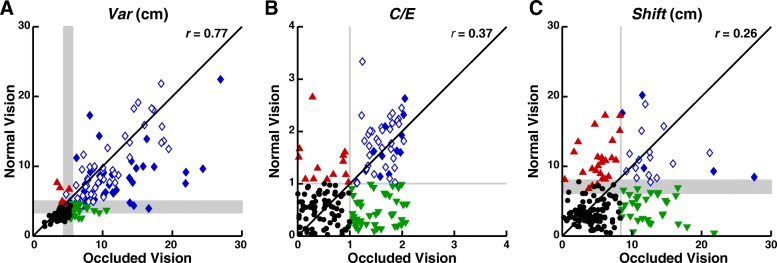
Relationships between measures of position sense with occluded and normal vision. Plots show the relationships for *Var* (**a**), *C/E* (**b**) and *Shift* (**c**). Black circles show subjects who were Normal in both conditions. Green triangles show subjects who showed Full compensation with normal vision. Blue diamonds show subjects who were Impaired in both conditions. Closed blue diamonds indicate subjects who exhibited Partial compensation and open blue diamonds indicate subjects who displayed Absent compensation. Red triangles show subjects who showed Inverse compensation (Normal with occluded and Impaired with normal vision). Grey shaded areas indicate areas in which performance classification (Impaired or Normal) varied with age, such that one extremity defined the boundary for a 20-year-old subject and the other extremity defined the boundary for a 90-year-old subject

Although many stroke survivors exhibited Full compensation, most displayed Partial or Absent compensation (Table 4). Figure 3a illustrates that *Var* showed a strong relationship between occluded and normal vision (Pearson correlation: *r =* 0.77, *p <* 10^− 12^; Fisher’s test: *p <* 10^− 9^), in which most subjects were either Normal (black circles; *n* = 55, 31%) or Impaired in both conditions (blue diamonds; *n* = 93, 53%). Notably, 116 stroke survivors (66%) were Impaired on *Var* with occluded vision and only 23 (20%) showed Full compensation (green triangles). In contrast, 37 (32%) exhibited Partial compensation (filled blue diamonds) and 56 (48%) displayed Absent compensation (open blue diamonds). Of the subjects in the Absent group, four (3%) were significantly worse with normal vision. Six subjects (3%) exhibited Inverse compensation (red triangles).

**Table 4:** Compensation for impaired position sense with normal vision

Measure	Factor	Group	Subjects	Impaired with OV	Compensation with NV		
					Full	Partial	Absent
*Var*		All subjects	177	116 (66%)	23 (20%)	37 (32%)	56 (48%)
	More affected side of body	Left-affected	108	87 (81%)	13 (15%)	29 (33%)	45 (52%)
		Right-affected	69	29 (42%)	10 (34%)	8 (28%)	11 (38%)
	Vascular Territory	MCA	117	79 (68%)	14 (18%)	28 (35%)	37 (47%)
		PCA	22	16 (73%)	5 (31%)	3 (19%)	8 (50%)
		ACA	6	2 (33%)	0 (0%)	1 (50%)	1 (50%)
		Brainstem	14	9 (64%)	1 (11%)	3 (33%)	5 (56%)
		Cerebellar	3	1 (33%)	0 (0%)	1 (100%)	0 (0%)
	Perceptual Impairment	Spatial Neglect	23	21 (91%)	2 (10%)	8 (38%)	11 (52%)
		Visual Defect	13	6 (46%)	0 (0%)	1 (17%)	5 (83%)
		Both	19	19 (100%)	1 (5%)	5 (26%)	13 (68%)
		Neither	122	70 (57%)	20 (29%)	23 (33%)	27 (39%)
*C/E*		All subjects	177	91 (51%)	42 (46%)	8 (9%)	41 (45%)
	More affected side of body	Left-affected	108	66 (61%)	26 (39%)	6 (11%)	34 (52%)
		Right-affected	69	25 (36%)	16 (64%)	2 (8%)	7 (28%)
	Vascular Territory	MCA	117	63 (54%)	28 (44%)	3 (5%)	32 (51%)
		PCA	22	16 (73%)	10 (63%)	2 (13%)	4 (25%)
		ACA	6	2 (33%)	1 (50%)	0 (0%)	1 (50%)
		Brainstem	14	1 (7%)	0 (0%)	0 (0%)	1 (100%)
		Cerebellar	3	2 (67%)	2 (100%)	0 (0%)	0 (0%)
	Perceptual Impairment	Spatial Neglect	23	20 (87%)	3 (15%)	2 (10%)	15 (75%)
		Visual Defect	13	5 (38%)	2 (40%)	1 (20%)	2 (40%)
		Both	19	15 (79%)	4 (27%)	2 (13%)	9 (60%)
		Neither	122	51 (42%)	33 (65%)	3 (6%)	15 (29%)
*Shift*		All subjects	177	52 (29%)	32 (62%)	3 (6%)	17 (33%)
	More affected side of body	Left-affected	108	38 (35%)	19 (50%)	3 (8%)	16 (42%)
		Right-affected	69	14 (20%)	13 (93%)	0 (0%)	1 (7%)
	Vascular Territory	MCA	117	39 (33%)	23 (59%)	3 (8%)	13 (33%)
		PCA	22	4 (18%)	3 (75%)	0 (0%)	1 (25%)
		ACA	6	2 (33%)	1 (50%)	0 (0%)	1 (50%)
		Brainstem	14	3 (21%)	3 (100%)	0 (0%)	0 (0%)
		Cerebellar	3	0 (0%)	0 (0%)	0 (0%)	0 (0%)
	Perceptual Impairment	Spatial Neglect	23	11 (48%)	5 (45%)	0 (0%)	6 (55%)
		Visual Defect	13	3 (23%)	0 (0%)	1 (33%)	2 (67%)
		Both	19	11 (58%)	6 (55%)	1 (9%)	4 (36%)
		Neither	122	27 (22%)	21 (78%)	1 (4%)	5 (19%)

*OV* occluded vision, *NV* normal vision

#### Group data - Contraction/Expansion

Compared to *Var*, fewer stroke survivors were Impaired on *C/E* with occluded vision and more exhibited Full compensation (Table 4). Figure 3b illustrates that *C/E* exhibited a moderate relationship between occluded and normal vision (Pearson correlation: *r =* 0.37, *p <* 10^− 6^; Fisher’s test: *p <* 10^− 9^). Most stroke survivors were either Normal (black circles, *n* = 73, 41%) or Impaired in both conditions (blue diamonds, *n* = 49, 28%). In contrast to *Var*, of the 91 stroke survivors (51%) who were Impaired on *C/E* with occluded vision, 42 (46%) showed Full compensation (green triangles), eight (9%) showed Partial compensation (filled blue diamonds), and 41 (45%) exhibited Absent compensation (open blue diamonds). Of the subjects in the Absent group, four (4%) performed significantly worse with normal vision. Thirteen subjects (7%) exhibited Inverse compensation.

#### Group data - Shift

Compared to *Var* and *C/E*, fewer stroke survivors were Impaired on *Shift* and more exhibited Full compensation (Table 4). Figure 3c illustrates that *Shift* exhibited a weak relationship between occluded and normal vision (Pearson correlation: *r =* 0.26, *p <* 10^− 3^; Fisher’s test: *p =* 0.003). Most stroke survivors were either Normal in both conditions (*n* = 100, 56%) or Impaired in both conditions (blue diamonds, *n* = 20, 11%). Although few stroke survivors (*n* = 52, 29%) were Impaired on *Shift* with occluded vision, 32 (62%) showed Full compensation (green triangles), three (6%) exhibited Partial compensation (filled blue diamonds), and 17 (33%) displayed Absent compensation (open blue diamonds; *t*-tests, *p ≥* 0.05). Of the subjects in the Absent group, two (5%) performed significantly worse with normal vision. Twenty-five subjects (14%) exhibited Inverse compensation (red triangles).

### Relationships of stroke features and perceptual impairments with compensation

#### Examining the impact of body side affected and stroke location

Both stroke features and perceptual impairments showed relationships with compensation (Table 4). Stroke survivors with a left-affected body side were more likely to exhibit impaired position sense with occluded vision and were more likely to display Partial or Absent than Full compensation. In contrast, none of the vascular territories exhibited a frequent pattern of compensation, although many individuals with MCA and PCA strokes displayed Impaired position sense with occluded vision.

#### Examining the impact of perceptual impairments

To investigate relationships between perceptual impairments and compensation, we examined compensation in stroke survivors with only *Visuospatial Neglect* (*n* = 23), only *Visual Field Defects* (*n* = 13), *Both* (*n* = 19), or *Neither* (*n* = 122). Individuals with only visuospatial neglect or both perceptual impairments were more likely to exhibit Impaired position sense with occluded vision and less likely to show Full compensation. We also observed weak correlations between BIT scores and improvements in *Var* and *C/E* (Table 5), although both *Var* and *C/E* exhibited higher correlations with FIM scores in both conditions individually.

**Table 5:** Relationships between robotic measures of position sense vision and clinical measures of function and impairment in stroke survivors who were *Impaired* with occluded vision.

Clinical Measure	Improvement			Occluded Vision			Normal Vision		
	*Var* (*n* = 116)	*C/E* (*n* = 91)	*Shift* (*n* = 52)	*Var* (*n* = 116)	*C/E* (*n* = 91)	*Shift* (*n* = 52)	*Var* (*n* = 116)	*C/E* (*n* = 91)	*Shift* (*n* = 52)
FIM	−0.05 *	−0.04 *	−0.11 *	−0.37 ‡	−0.34 †	−0.20 *	−0.36 ‡	−0.18 *	−0.07 *
BIT	−0.16 *	−0.32 ‡	−0.19 *	−0.46 §	−0.32 †	−0.07 *	−0.63 §	−0.45 ‡	−0.34 †
PPB more affected	−0.20 *	−0.17 *	−0.08 *	−0.01 *	−0.25 †	−0.05 *	−0.17 *	−0.28 †	−0.03 *
PPB less affected	−0.02 *	−0.02 *	−0.14 *	−0.33 ‡	−0.37 ‡	−0.21 *	−0.30 ‡	−0.14 *	−0.02 *
MoCA	−0.09 *	−0.29 †	−0.21 *	−0.16 *	−0.18 *	−0.04 *	−0.29 †	−0.33 †	−0.27 *

Spearman correlations between clinical scores (FIM, BIT, PPB, MoCA and improvements/measures of position sense. Symbols indicate significance of one-tailed tests for improvements (positive relationships) and measures (negative relationships): * *p* < 0.05, † *p* < 0.01, ‡ *p*< 0 .001, § *p* < 10^-6^

### Relationships between compensation and functional performance

**Fig. 4:**
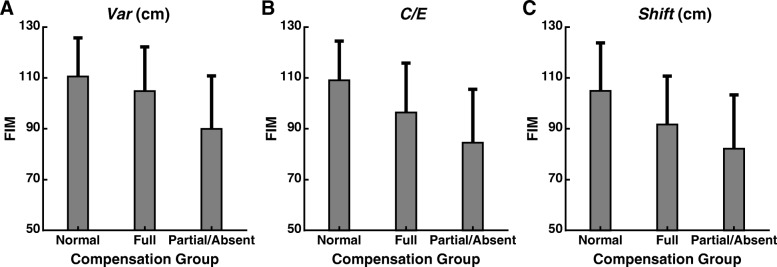
Functional performance exhibited by different compensation groups. Plots show the mean FIM (±SD) for stroke survivors classified by *Var* (**a**), *C/E* (**b**), and *Shift* (**c**) into three compensation groups: Normal, Full, and Partial/Absent combined

We did not observe an association between improvements in position sense and greater functional independence (Table 5). Notably, none of our measures of position sense displayed improvements that were correlated with FIM scores. However, *Var* and *C/E* were correlated with FIM scores in both conditions individually. We also compared FIM scores of stroke survivors placed in the Normal, Full, and Partial/Absent categories (Fig. 4). Whether we used *Var*, *C/E*, or *Shift* to categorize subjects, the Normal group consistently displayed the highest FIM scores, followed by the Full group with intermediate FIM scores, and the Partial/Absent compensation group with the lowest FIM scores (one-way ANOVAs, all *p* < 0.001).

### Relationships between compensation and measures of motor and cognitive impairment

Our final analyses examined the extent to which compensation was associated with motor and cognitive impairments (Table 5). Smaller improvements on *Var* were weakly associated with decreased dexterity of less affected hand (i.e., the hand moved during matching) on the PPB. Furthermore, *Var* with normal vision and *C/E* with both occluded and normal vision were weakly correlated with dexterity of less affected hand on the PPB. We also found that dexterity of more affected hand on the PPB was moderately correlated with *Var* in both conditions and *C/E* with occluded vision. Finally, MoCA scores exhibited weak correlations with improvements in *C/E*, with *Var* and *C/E* with occluded vision, and with all three measures with normal vision.

## Discussion

Our first objective was to determine the extent to which stroke survivors can use vision to compensate for impaired position sense. Surprisingly, many stroke survivors could not use vision to fully compensate for impairments on all three measures of position sense. While several subjects exhibited Partial compensation, improvements were insufficient to achieve comparable performance to controls. Many stroke survivors also exhibited Absent compensation. Overall, these findings refute our hypothesis that most stroke survivors would exhibit better position sense with vision.

Our second objective was to examine the extent to which stroke features and perceptual impairments are associated with differences using vision to compensate for impaired position sense. Individuals with right-sided cerebral strokes, particularly those with visuospatial neglect, displayed limited compensation for impaired position sense. Future research should investigate whether improvements in visuospatial neglect over time are coupled with increases in the ability to use vision to compensate for proprioceptive impairments. Understanding the underlying mechanisms behind such potential coupling could help to increase our understanding of the impact of attention, eye movements and visuospatial function on proprioceptive processing.

Our third objective was to examine the extent to which compensation for impaired position sense is associated with increased functional ability. Greater compensation was not associated with increased functional independence, although individuals who exhibited Full compensation with vision tended to have greater functional independence than those with Partial or Absent compensation. Better position sense with both occluded and normal vision was also associated with increased functional ability. Developing other treatments for proprioceptive impairments may help to further improve functional outcomes after stroke.

### Role of vision in compensating for impaired position sense

Our findings raise several important questions. Why are many stroke survivors unable to use vision to compensate for impaired position sense? If patients with peripheral nerve lesions can use vision to compensate for impaired position sense, what is different about stroke? Does stroke interfere with low-level processing of proprioceptive or visual information, higher-order integration of proprioceptive and visual information, and/or cognitive and motor functions used for planning and execution of our task?

Both impaired position sense and poor compensation were strongly associated with visuospatial neglect, suggesting that visuospatial awareness resulting from higher-order integration of vision and proprioception is crucial for compensation. However, compensation was also greater on *C/E* and *Shift*, which involve higher-order visuospatial awareness. These findings suggest that both low-level and higher-order integration of vision and proprioception may contribute to compensation for impaired position sense.

Both cognitive impairments and motor impairments of the less-affectedupper-limb were weakly associated with compensation, suggesting that they may have influenced our results. Since we did not complete a comprehensive cognitive assessment, we cannot ascertain whether some cognitive impairments exert greater interference on compensation than others. We have recently developed novel methods for examining cognitive organization of eye movements with our robotic device [42, 43] and have demonstrated that impaired organization of eye movements is associated with difficulties performing tasks involving reaching, hand function and mobility [43, 44]. However, the current study did not examine whether impaired organization of eye movements interfered with collecting visual information used for compensation.

Many stroke survivors with right-sided MCA and PCA territories exhibited Partial or Absent compensation. Recent studies have examined the neural correlates of position matching using various techniques such as task-based functional MRI [45], electroencephalography [46], voxel-based lesion symptom mapping [47] and statistic ROI mapping [48]. All of these studies, have in some way, indicated the importance of association areas (eg. supramarginal gyrus, Heschl’s gyrus) in the performance of position matching. We put forward that the poor compensation we observed in the present study may reflect disrupted integration of visual and proprioceptive information within parietal and frontal areas associated with impaired position sense and kinesthesia with occluded vision [47, 49]. The human superior parietal lobe is thought to mediate sensorimotor integration [50] and attention to both visuospatial [51–53] and kinesthetic stimuli [54, 55]. Electrophysiological studies in non-human primates have also found neurons in parietal cortex that combine visual and hand position signals [56]. Furthermore, hand position modulates saccadic eye movements in the lateral intraparietal area [57] and frontal eye fields [58], and eye position signals have been recorded in Area 3a [59], suggesting that organized eye movements are important for integrating visual and proprioceptive information. Overall, these studies agree with the suggestion that information from many brain areas is integrated to form a representation of the outside world [60]. Given that a stroke can produce damage and/or disconnection of several brain regions that process visual or proprioceptive information, we hypothesize that damage and/or disconnection of several parietal and frontal areas could interfere with compensation by disrupting integration of proprioception and vision.

### Implications for stroke rehabilitation

Our findings have important implications for stroke rehabilitation clinicians. For stroke survivors who are able to compensate for impaired position sense with vision, this rehabilitation approach is reasonable. However, this approach may not be effective for stroke survivors who exhibit Absent compensation. Given the importance of proprioception for functional movement [1, 2], this highlights the need to develop and rigorously test new interventions for rehabilitation of the proprioceptive system. Robotics [61–63] and other techniques (eg. electrical stimulation [15, 64]) have demonstrated some ability to improve proprioception following stroke. Recently, a study in healthy adults using vibrotactile stimulation [65] demonstrated that this is yet another technique that might be useful in enhancing proprioception. While the results of investigations into enhancing proprioception have demonstrated encouraging results, we expect larger controlled trials in stroke will be necessary before we see widespread clinical adoption of these novel techniques [10]. It is also unclear whether vision can be used to promote rehabilitation in individuals with Partial compensation. With practice, these individuals may learn to use vision to fully compensate for impaired position sense or they may continue to exhibit incomplete compensation because the mechanisms that underlie learning have been disrupted. Additional studies are needed to resolve these alternative hypotheses.

Caution must also be taken before applying our results to proprioceptive impairments affecting the lower extremity. In contrast to our results, a previous study found that chronic stroke survivors can use vision to compensate for proprioceptive impairments that interfere with successful obstacle clearance during gait [66]. This difference may be explained by the fact that our study examined behavior during a perceptual task and the gait study examined a visuomotor task involving obstacle avoidance.

## Limitations

The present study examined the ability to use vision to compensate for impaired position sense of the proximal upper extremity during sub-acute stroke. This timing was chosen because individuals were engaged in inpatient stroke rehabilitation at the time of recruitment. However, experience-dependent improvements in compensation with vision might produce different results at later time periods. Second, we examined position sense of the proximal upper extremity using a robotic exoskeleton constrained to the horizontal plane. Although position sense in 3D space might yield different interactions with vision [67], we would expect to observe similar results because position sense in 3D space would largely arise from muscle spindles embedded in the same shoulder and elbow muscles [68]. However, compensation might be different at the distal upper extremity because of the distinct underlying musculature and our greater use of vision for object manipulation [69]. Third, we carried out a rudimentary analysis of the relationships between compensation and vascular territories. Detailed neuroimaging analyses of relationships with lesions (MRI), structural disconnection (DTI) and functional disconnection (resting-state fMRI) may provide additional information for better prognosis of who will benefit from different treatments, including visual compensation. Lastly, our correlation analyses did not causally test whether greater compensation with vision produces improvements in controlling upper extremity movements and performing functional activities. Randomized controlled trials are needed to test these causal relationships and obtain greater insight into the potential impact on daily living.

## Conclusion

Many stroke survivors are unable to compensate for impaired position sense using vision. This contradicts traditional thinking that vision can be used to compensate for post-stroke impairments of proprioception. It also highlights the need to develop other techniques for improving proprioceptive function in individuals with sensory loss following stroke.

## Data Availability

The dataset analyzed in the current study is available upon request from the corresponding author upon reasonable request.

## Abbreviations

ACA: Anterior Cerebral Aretery
ANOVA: Analysis of Variance
BIT: Behaviourial Inattention Test
*C/E*: Contraction/expansion
CMSA: Chedoke McMaster Stroke Assessment
FIM: Functional Independence Measure
MAS: Modified Ashworth Score
MCA: Middle Cerebral Artery
MoCA: Montreal Cognitive Assessment
PCA: Posterior Cerebral Artery
PPB: Purdue Pegboard
*Shift*: Systematic shift
TLT: Thumb Localizer Test
*Var*: Variability
